# Exploring trajectories of depressive symptoms in North Korean defectors: A latent class mixed analysis

**DOI:** 10.3389/fpsyt.2022.943803

**Published:** 2022-08-30

**Authors:** Dahae Kim, Sohee Park, Ji Hyun An, Myung Hyun Kim, Hyein Chang, Jin Pyo Hong

**Affiliations:** ^1^Department of Psychiatry, Samsung Medical Center, Seoul, South Korea; ^2^Department of Psychiatry, Samsung Medical Center, Sungkyunkwan University School of Medicine, Seoul, South Korea; ^3^Department of Psychology, Sungkyunkwan University, Seoul, South Korea

**Keywords:** depression, trajectories, anxiety, mental health, North Korean defector

## Abstract

**Background:**

North Korean defectors (NKDs) are known to be vulnerable to depression due to the influence of various psychosocial factors during their settlement process. Therefore, this study aimed to explore different trajectories of depressive symptoms in NKDs and identify predictors of the worsening of depressive symptoms. In particular, the focus was on whether posttraumatic stress disorder (PTSD) functions as a significant predictor.

**Methods:**

Participants consisted of 300 NKDs who settled in South Korea within 3 years of entering in the country. Five waves of longitudinal data collected from 2016 to 2019 were used. In this study, the North Korean Composite International Diagnostic Interview (NK-CIDI), a diagnostic interview tool, was conducted at baseline and depressive symptoms were measured at each wave. Trajectory identification was based on latent class mixed modeling. Logistic regression was used to extract the significant factors predicting a high/increasing depressive symptom trajectory.

**Results:**

Two trajectories of depressive symptoms were identified: “low/stable symptom” (76.7% of participants) and “high/increasing symptom” (23.3% of participants). Predictors of the high/increasing symptom group were being female and a low use of mental health services. Generalized anxiety disorder, social phobia, and alcohol dependence acted as significant predictors. Although PTSD was not a significant predictor, self-reported PTSD symptom scores were high in the high/increasing symptom group.

**Conclusions:**

Identifying the trajectories of depressive symptoms in NKDs helps identify the risk factors of clinically vulnerable groups. In the process of establishing government-level interventions, it may be helpful to create a therapeutic environment. In addition, when evaluating initial psychiatric problems, it is important to primarily consider NKDs' anxiety levels and alcohol problems. There should also be an appropriate consideration of PTSD symptoms.

## Introduction

North Korean defectors (NKDs) have been stably entering South Korea since the 2000s. Although their numbers have dropped sharply, due to COVID-19, they are still entering the country in significant numbers every year. As part the statistics of 2021, the number of NKDs residing in South Korea was estimated to be 33,800. Consequently, their settlement problems have emerged as an important issue in South Korea. According to many studies, various stress factors, such as cultural differences, loneliness, and unemployment, make it difficult for them to adapt to South Korean society (1). Therefore, they appear to be a group that is vulnerable to mental health issues. According to a meta-study of refugees in Western countries, about 10% of them were diagnosed with post-traumatic stress disorder (PTSD) and 5% with major depressive disorder (2). Several studies on NKDs' mental health have shown that a significant number of these individuals have depressive symptoms (3). In addition, studies conducted in South Korea have shown that more than 40% of the NKDs have accompanied symptoms of depression and somatization (4). In terms of depression, the general population has a prevalence of 4.9%, while the NKDs have a relatively high prevalence of 22.3% (5). In particular, studies reported that NKDs living in South Korean society were more exposed to discrimination experiences than those living in other countries (6, 7). In addition, influenced by the system-adaptive attiude in totalitarian society, there is a tendency to be passive in properly reporting symptoms and receiving treatment (6). Therefore, the depression level of NKDs is a factor that should be managed. In addition, the level of psychological adaptation can be indirectly identified through the severity of depressive symptoms (8). Accordingly, many refugee studies have used these symptoms as a key indicator of refugee groups' mental health (9). In other words, using the severity of depressive symptoms as a mental health indicator is essential to determine adaptability and establish an appropriate support plan.

Refugee status is often determined by specific external events, such as political factors, rather than individual will. It is characterized by a high fluctuation in individuals' mental health issues depending on the extent of infrastructure in the new country (10). Therefore, in addition to individual predisposition, understanding the environmental factors surrounding refugees is essential. The more stress-generating life events occur in genetically vulnerable individuals, the higher the likelihood that depression will worsen. It is important to specifically explore “environmental toxins” that exacerbate depressive symptoms of NKDs (11, 12). For example, factors that hinder social adaptation in South Korean were insufficient employment opportunities, social prejudice of South Koreans, language barrier, and cultural differences. In addition, according to the survey in 2021, many NKDs showed loneliness due to an environment separated from their families (13). Furthermore, a study that measured the severity of depression, demographic variables, and personal variables of 160 NKDs with an average residence period of more than seven years in South Korea, analyzing the correlation between variables, found that many factors such as educational year or marriage status could worsen depressive symptoms (14). According to a meta-analysis of the literature over a decade, studies reported that low social support (15, 16) and low use of mental health services (10, 17) were related to these symptoms. Demographic variables including age, gender, education level (18–20), socioeconomic status (21, 22), and psychological variables (e.g., resilience, perceived life satisfaction, and loneliness) also play a role (22, 23). As a solution, refugee studies claimed that creating a therapeutic environment with psychosocial and drug interventions can engender mental health improvements (10, 17).

Trajectories of depressive symptoms were predicted by the presence of psychiatric disorders (24). Some researchers explored the relationship between PTSD, and mental health problems. One study revealed that PTSD was a major mediator between trauma exposure and comorbid psychiatric symptoms through structural equation modeling with 144 NKDs (25). Additionally, a study on 74 NKD youths showed a strong relationship between PTSD and depression using path analysis (26). Moreover, PTSD was significantly associated with depression symptom severity. However, both these studies targeted the youth and had a small sample size, making it difficult to generalize the findings for NKDs of all ages. In addition, the diagnosis of PTSD was based on self-report results. It was also difficult to determine causality as the studies were cross-sectional. Thus, previous studies that examined the effect of PTSD on NKDs' depressive symptoms were insufficient. In addition to PTSD, anxiety or alcohol-related disorders also fuction as predictors of depressive symptoms (27, 28), but most of studies did not consider the presence of psychiatric disorders other than PTSD.

However, longitudinal studies identifying variables that affect depressive symptoms are limited among NKDs (3). Some researchers in South Korea have attempted to explore the trajectories of depressive symptoms, but they have found it difficult to conduct longitudinal designs. For example, in a previous study, after comparing depressive symptoms between two waves, individuals were classified into alleviated, deteriorated, and prolonged groups according to the degree of change in depressive symptoms (8). The deteriorated and prolonged depression groups were more likely to have lower resilience. However, the long-term trajectory could not be examined due to the short follow-up periods. Moreover, using the Center for Epidemiologic Studies Depression Scale (CES-D), it was found that depressive symptoms had an increasing trajectory, with no significant difference based on gender and age (29). Despite their important findings, studies could not identify the subgroups that were expected to exhibit differences in the course of developing symptoms, as they only explored the unitary trajectory of depressive symptoms (30, 31). On the other hand, based on the need for a longitudinal perspective, a recent refugee study identified patterns of changes in depressive/PTSD trajectories classified into four classes using a latent transitional model. It suggested that post-migration stressors, such as discrimination and separation from parents, played a major role in developing psychopathology (32). Thus, it is essential to investigate different trajectory patterns in NKDs that may be associated with different etiological processes, comorbidities, and psychosocial outcomes (24).

Accordingly, this longitudinal study aimed to explore different trajectories of depressive symptoms through latent class mixed analysis. This assessment included the intrapersonal, demographic and psychological variables known to be associated with depressive symptoms. Finally, this study also aimed to identify the predictors of the aforementioned trajectories and explore whether PTSD can predict increasing symptom trajectories, especially among several mental disorders.

## Materials and methods

### Participants and procedures

This research was a cohort study on NKDs, conducted with the support of the Ministry of Health and Welfare of Korea. Among the NKDs registered at Hana Center (which is a regional adaptation center), the sample comprised those who entered South Korea within the last 3 years before the first assessment. A total of 300 adults, aged between 18 to 73 years, voluntarily agreed to participate in this research after being informed of its purpose. The assessment was conducted every 6 months from 2016 to 2019. Data from five waves (T1; 06.2016, T3; 01.2017, T5; 06.2018, T6; 11.2018, and T7; 03.2019), which measured depressive symptoms among seven waves, were used for analysis. Specifically, of the original 300 people who participated in this study, the follow-up session included 199 from wave three, 189 from wave five, and 172 from waves six and seven. In the first assessment, a structured diagnostic interview was performed face to face. After the interview, the participants filled out self-report questionnaires related to mental health. All the questionnaires used in the analysis were conducted together in the first assessment. The follow ups were conducted with online self-report questionnaires. This study was approved by the Institutional Review Board of Samsung Medical Center (SMC 2015-05-042-002).

### Instruments

Depressive symptoms were measured by The North Korean version of the CES-D (CES-D-NK) (33). CES-D-NK was translated (34) and validated into North Korean (35). It comprises a four-point Likert scale, ranging from “rarely or never (0)” to “most or all of the time (3),” with a potential total score of 0 to 60. The higher the score, the more severe the depressive symptoms. Individuals with points ranging from 16 to 25 were classified as having mild depressive symptoms, and those with more than 25 points were considered to have clinically significant depressive symptoms (36). The CES-D was used for all five waves. The Cronbach's alpha was 0.931–0.956.

### Mental disorders and suicidal ideation

In addition to the socio-demographic variables (i.e., sex, age, age of defection, education year, marriage status, number of children, and household income), the North Korean Composite International Diagnostic Interview (NK-CIDI) was used to measure mental illnesses. The CIDI is a structured diagnostic interview tool that employs diagnostic criteria from the Diagnostic and Statistical Manual of Mental Disorders, fourth edition (DSM-IV), and is commonly used in large-scale epidemiological studies (37). North Korean language scholars and mental health experts translated the NK-CIDI tool, while considering the sociocultural background of North Korea, and clarified its reliability and validity (38). The results were analyzed with a focus on PTSD, generalized anxiety disorder, panic disorder, specific phobia, alcohol dependence, and alcohol abuse. In addition, lifetime suicidal ideation was measured based on “yes” responses to “Have you ever seriously thought of dying by suicide? which is included in the NK-CIDI module.

### Psychological characteristics

The North Korean version of the Impact of Event Scale-R (IES-R-NK) measured trauma-related symptoms (39). A revised edition was subsequently devised to reflect the DSM-IV PTSD diagnostic criteria (40) and was translated and validated into North Korean (41). The associated five-point Likert scale, ranging from “not at all (0)” to “extremely (4),” can produce total scores between 0 and 88. The higher the score, the higher the severity of the symptoms. Individuals with points ranging from 0–24 were classified as normal, those with 25–39 points as mild to moderate, and those with more than 40 points as severe (42).The Cronbach's alpha was 0.959. Furthermore, the Korean version of the University of California, Los Angeles (UCLA) loneliness scale measured the degree of subjective loneliness (43). It was translated and validated into Korean (44). It contains a four-point Likert scale ranging from “not at all (1)” to “frequently (4),” with a total score range of 10 to 40. The higher the score, the higher the subjective loneliness. The Cronbach's alpha was 0.875. Finally, the Korean version of the Conner-Davidson Resilience Scale (CD-RISC) measured the degree of resilience (45). It was translated into Korean (46). This study used a version translated into North Korean by experts. It operates on a five-point Likert scale, with total scores ranging from 0 to 100. The higher the score, the higher the resilience. The Cronbach's alpha was 0.947.

### Perceived social support and utilization of mental health services

Two items were used to measure the degree of perceived social support. The first question, “How much psychological support do you currently receive from your family, relatives, friends, and others around you?,” was used to measure psychological support. This question measures the degree of support related to emotional expression such as affection, love, and empathy. The second question, “How much practical support do you currently receive from your family, relatives, friends, and others around you?” was used to measure practical help. This question measures the degree of support associated with instrumental assistance, such as goods or services. Both questions were answered on a 10-point scale ranging from “none (1)” to “enough (10).” Each item was considered individually without adding them up. Moreover, we adopted the social capital question from the Legatum Prosperity Index (47). Among the seven questions, we used “Have you attended a place of worship or religious service within the last seven days?” to measure religious support (“yes” or “no” responses). Finally, the WHO's World Mental Health questionnaires were used to determine the use of mental health services objectively (48). We specifically employed questions related to whether or not the individuals had discussed mental health problems with experts in their lifetime, such as psychiatrists, clinical psychologists, and other experts (“yes” or “no” responses) (49).

### Statistical analysis

A latent growth mixture model analysis was conducted to determine the trajectory of NKDs' depressive symptoms. Using data from five waves of CES-D-NK scores (T1, T3, T5, T6, and T7), we applied a linear function to determine the most suitable number of trajectories and fit indices to ascertain the optimal number. We also employed the Akaike information criterion (AIC) (50) and Bayesian Information Criterion (BIC) (51), for which the smaller the value, the better the suitability and simplicity. In addition, we considered the Lo-Mendell-Rubin value, which compares and verifies the relative suitability of k-1 and k models, in combination with the entropy value: the closer the value is to 1, the better the quality (52). We used MPLUS 7 for analysis and full information maximum likelihood for missing values. Logistic regression was employed to extract the factors predicting high/increasing depressive symptom trajectory. The predictors were all variables measured at baseline, and the resulting variables were two projected groups (0 = low/stable symptom, 1 = high/increasing symptom). In addition to the factors extracted as predictors, *t*-test and chi-square analyses were conducted to explore the difference between groups of individual variables. The analyses were performed using SPSS version 25 (IBM, Armonk, NY, USA). The skewness and kurtosis of the continuous variables were <2, satisfying the assumption of normality.

## Results

### Sample characteristics

Table 1 presents the participants' descriptive statistics. The mean age at baseline was 38.73 (± 11.9) years, and 78.3% of the participants were women. A total of 71.3% of participants were unemployed and more than 90% reported having low to middle income. At baseline, the CES-D-NK score was 13.88 points (SD: 12.64), and the IES-R-NK score was 28.86 points (S.D: 20.27). **Table 4** presents the ratio of major mental disorders based on DSM-IV criteria. Around 24.3% of participants met the criteria for major depressive disorder, followed by 22% for social phobia, 15.3% for PTSD, and 10.7% for alcohol dependence.

**Table 1:** Sociodemographic and psychological factors of participants at baseline.

	**Total (*n* = 300)**
**Female sex**, ***n*** **(%)**	235 (78.3%)
**Age, years, M (SD, range)**	38.73 (11.9, 18–73)
**Age of defection, M (SD, range)**	37.45 (11.81, 16–73)
**Education, years, M (SD, range)**	11.02 (2.37, 0–20)
**Marriage status**, ***n*** **(%)**	
Married	99 (33%)
Others	201 (67%)
**Child**, ***n*** **(%)**	
0	95 (31.7%)
1	111 (37%)
2 ≤	94 (31.3%)
**Occupational status**, ***n*** **(%)**	
Yes	86 (28.7%)
No	214 (71.3%)
**Household income**, ***n*** **(%)**	
Under $400^a^	107 (35.7%)
$400-$2,400^b^	179 (59.7%)
$2,400 above	7 (2.3%)
Non-response	7 (2.3%)
**Mental health service utilization**, ***n*** **(%)**	
**Yes**	226 (75.3%)
**No**	74 (24.7%)
**Religious life**, ***n*** **(%)**	
**Yes**	144 (48%)
**No**	156 (52%)
**Lifetime suicidal ideation**, ***n*** **(%)**	65(21.7%)
**Baseline psychological index**	
IES-R-NK, M (SD)	28.86 (20.27)
CES-D-NK, M (SD)	13.88 (12.64)
UCLA loneliness, M (SD)	17.88 (5.15)
CD-RISC, M (SD)	72.5 (18.32)
Social support, M (SD) Psychological support	6.20 (2.82)
Practical help	5.47 (2.98)

M, Mean; SD, Standard Deviation.

^a^about five hundred thousand Korean won, ^b^about three million Korean won. IES-R-NK, Impact of Event Scale Revised–North Korea Scale; CES-D-NK, North Korean version of the Center for Epidemiological Studies–Depression Scale; CD-RISC, Connor–Davidson Resilience Scale.

### Latent class identification

**Figure 1:**
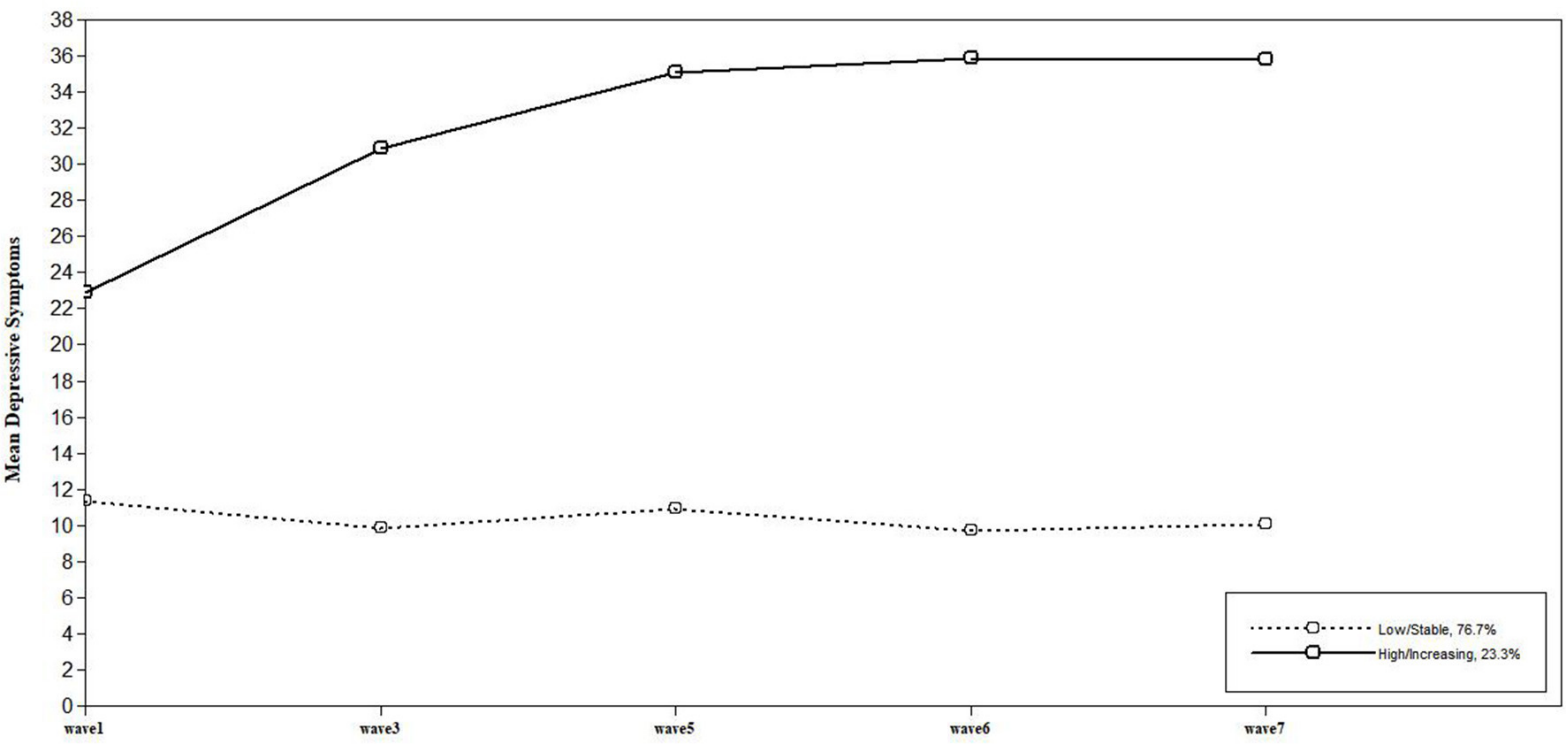
Two trajectories of depressive symptoms: Class 1. low/stable symptom (76.7%), Class 2. high/increasing symptom (23.3%). Based on the data of five waves, average depression was measured as the CES-D-NK score.

Table 2 reveals the fit indices of three class models. Among these, the model with three trajectories showed relatively low AIC and BIC values, as well as high entropy values. However, due to its low sample size, this model could not be used for further analysis. Thus, based on the number of participants and fit indices, we used the model with two trajectories. Figure 1 presents the mean of CES-D-NK scores for the two trajectories. The first class, labeled the “low/stable symptom” group (N = 236; intercept = 11.022, *p* < 0.001; slope = −0.315, *p* < 0.001), reflected minimal levels of depression, maintained without a significant change in symptoms over time. The second class, labeled the “high/increasing symptom” group (N = 64; intercept = 25.650, *p* < 0.001; slope = 3.200, *p* < 0.001), showed progressively worsening depressive symptoms at a moderate level.

**Table 2:** Fit indices for latent class trajectory models for NKDs' depressive symptoms.

**Classes**	**AIC**	**BIC**	**LogLikelihood**	**Entropy**	**LoMendel**	**Least class predicted *N* (%)**
1	8,266.718	8,292.645	−4,126.359			
2	7,919.824	7,956.862	−3,949.912	0.761	−4,126.359***	64 (22.3%)
3	7,150.99 5	7,199.144	−3,562.598	0.682	−3,586.841***	9 (3%)

AIC, Akaike Information Criterion; BIC, Bayesian Information Criterion.

^***^p <0.001.

### Comparison of each class at baseine

Tables 3, 4 present the demographic and psychosocial variables, as well as mental disorder diagnosis rates between each class. In particular, in the high/increasing symptom group, the initial level of mental health symptoms was high, with trauma-related symptoms at a severe level (IES-R-NK: M = 40.28) and depressive symptoms at a mild to moderate level (CES-D-NK: M = 24.84). In the low/stable symptom group, traumatic and depressive symptoms were similar to or lower than each cut-off score. Based on the chi-square and *t*-tests, there were no clinically significant differences between classes with regard to age, educational background, income level, marital status, and degree of perceived social support. The high/increasing symptom group had a high proportion of women (χ^2^(1) = 11.39, *p* < 0.001), children [*t*_(298)_ = 2.18, *p* = 0.030], and unemployed individuals (χ^2^(1) = 6.76, *p* = 0.009), as well as a lower rate of health service utilization (χ^2^(1) = 18.66, *p* < 0.001). In addition, trauma-related symptoms [*t*_(298)_ = 5.41, *p* < 0.001], depressive symptoms [*t*_(298)_ = 8.43, *p* < 0.001], loneliness [*t*_(298)_ = 3.06, *p* = 0.002], and lifetime suicidal ideation [*t*_(298)_ = 5.95, *p* = 0.015] were significantly high, while resilience was low [*t*_(298)_ = 2.19, *p* = 0.031] (Table 3). The results also showed a higher prevalence of most mental disorders, excluding alcohol-related disorders (Table 4).

**Table 3:** Between-class comparison of sociodemographic and psychological factors at baseline.

**Characteristics**	**Low/stable symptom (*N* = 236)**	**High/increasing symptom (*N* = 64)**	**Significant test^a^**
**Gender**, ***n*** **(%)**			
Female	175 (74.2%)	60 (93.8%)	11.393**
Male	61 (25.8%)	4 (6.3%)	
**Age, M (SD)**	38.21 (11.82)	40.66 (12.10)	1.440
**Age of defection, M (SD)**	30.21 (13.31)	33.30 (14.60)	1.529
**Education, M (SD)**	11.13 (2.16)	10.61 (3.00)	1.303
**Child, M (SD)**	1.04 (1.02)	1.34 (0.85)	2.419*
**Occupational status**, ***n*** **(%)**			
Yes	76 (32.2%)	10 (15.6%)	6.767***
No	160 (67.8%)	54 (84.4%)	
**Household income**, ***n*** **(%)**			
Under $400^b^	81 (34.9%)	26 (42.6%)	7.41
**Marriage status**, ***n*** **(%)**			
Married	78 (33.1%)	21 (32.8%)	0.001
Others	158 (66.9%)	43 (67.2%)	
**Mental health service utilization**, ***n*** **(%)**			
Yes	191 (80.9%)	35 (54.7%)	18.662***
No	45 (19.1%)	29 (45.3%)	
**Religious life**, ***n*** **(%)** **Yes**	111 (47.4%)	33 (51.6%)	0.574
**Baseline psychological index**			
IES-R-NK, M (SD)	25.54 (18.72)	40.28 (21.44)	5.007***
CES-D-NK, M (SD)	11.09 (9.86)	24.84 (16.03)	8.438*
UCLA loneliness, M (SD)	17.41 (4.75)	19.61 (6.18)	3.065**
CD-RISC, M (SD)	74.27 (17.75)	68.46 (19.03)	2.169***
Social support, M (SD) Psychological support	5.54 (2.90)	5.20 (3.28)	0.742
Practical help	6.23 (2.73)	6.09 (3.18)	0.348
**Lifetime suicidal ideation**, ***n*** **(%)**	44 (18.6%)	21 (32.8%)	5.955*

M, Mean; SD, Standard Deviation.

^a^Means or proportions are compared with t-test or Chi-square tests. ^b^about five hundred thousand Korean won. IES-R-NK, Impact of Event Scale Revised–North Korea Scale; CES-D-NK, North Korean version of the Center for Epidemiological Studies–Depression Scale; CD-RISC, Connor–Davidson Resilience Scale.

^*^p <0.05.

^**^p <0.01.

^***^p <0.001.

**Table 4:** Between-class comparison of prevalence of DSM-IV major mental disorders at baseline.

**Psychiatric disorders, *n* (%)**	**Total (*N* = 300)**	**Low/Stable (*N* = 236)**	**High/Increasing (*N* = 64)**	**Significant test^a^**
Major depressive disorder	73 (24.3%)	43 (18.2%)	30 (46.9%)	22.452***
Dysthymia	17 (5.7%)	8 (3.4%)	9 (14.1%)	10.728**
Posttraumatic stress disorder	46 (15.3%)	29 (12.3%)	17 (26.6%)	7.902**
Generalized anxiety disorder	36 (12%)	21 (8.9%)	15 (23.4%)	10.078**
Panic disorder	16(5.3%)	5 (2.1%)	11 (17.2%)	22.643***
Social phobia	66 (22%)	8 (3.4%)	14 (21.9%)	25.316***
Specific phobia	22 (7.3%)	40 (16.9%)	26 (40.6%)	16.446***
Alcohol dependence	32 (10.7%)	21 (8.9%)	11 (17.2%)	3.630
Alcohol abuse	33 (11%)	22 (9.3%)	11 (17.2%)	3.182

^a^Proportions are compared with Chi-square test.

*p <0.05.

**p <0.01.

***p <0.001.

### Predictors of high/increasing symptom class

Table 5 presents the results of the logistic regression. The variance inflation factor (VIF) among independent variables showed no evidence of a multicollinearity problem (VIF = 1.031–1.815). Gender and health service utilization were predictors for a person belonging to the high/increasing symptom class. Specifically, female participants were found to have a higher risk of being classified in the increasing symptom class (OR = 12.046, CI = 2.508–57.863, *p* = 0.002), as well as those who had never discussed mental health problems with experts in their lifetime (OR = 0.274, CI = 0.118–0.633, *p* = 0.002). Generalized anxiety disorder (OR = 3.663, CI = 1.258–10.667, *p* = 0.017), social phobia (OR = 9.121, CI = 2.341–35.543, *p* = 0.001), and alcohol dependence (OR = 4.714, CI = 1.044–21.289, *p* = 0.044) diagnoses at baseline were also predictors of being in the high/increasing symptom class. However, PTSD did not significantly predict worsening depressive symptoms (OR = 0.404, CI = 0.116–1.408, *p* = 0.155).

**Table 5:** Logistic regression of factors predicting high/increasing depressive trajetory group.

	**OR**	**95% Cl**	***p*-value**
Gender (ref = male)			
Female	12.046	2.508–57.863	0.002
Age	1.029	0.995–1.064	0.101
Education	0.867	0.727–1.034	0.112
Child	1.454	0.966–2.190	0.073
Occupational status (ref = no)	0.632	0.191–2.097	0.454
Household income	0.914	0.654–1.277	0.599
UCLA loneliness W1	1.052	0.961–1.152	0.272
CD-RISC W1	0.993	0.969–1.018	0.576
Mental health service utilization (ref = no)	0.274	0.118–0.633	0.002
Psychological support	1.062	0.882–1.279	0.524
Practical help	0.992	0.846–1.163	0.918
Religious life (ref = no)	0.601	0.262–1.378	0.229
Marriage status	1.901	0.780–4.631	0.157
Posttraumatic stress disorder	0.404	0.116–1.408	0.155
Generalized anxiety disorder	3.663	1.258–10.667	0.017
Panic disorder	2.981	0.546–16.282	0.207
Social phobia	9.121	2.341–35.543	0.001
Specific phobia	1.625	0.623–4.240	0.321
Alcohol dependence	4.714	1.044–21.289	0.044
Alcohol abuse	3.987	0.876–18.140	0.074
Lifetime suicidal ideation (ref = no)	0.609	0.223–1.660	0.332

OR, odds ratio; CI, confidence interval.

CD-RISC, Connor–Davidson Resilience Scale.

## Discussion

To the best of our knowledge, this is the first study to identify the latent trajectories of depressive symptoms among NKDs over time using latent class mixed analysis. This study is meaningful in that it explored the trajectory of depression from 2016 to 2019, using 300 samples of NKDs. In addition to self-reporting questionnaires, face-to-face interviews were conducted with 300 NKDs by trained professionals, which contributed to improve diagnostic accuracy. This study aimed to explore the characteristics of each group and predictive factors of the high/increasing depressive symptom group. Our first main finding was that two trajectories were identified: “low/stable symptom” (76.7%), characterized by minimal depressive symptoms, and “high/increasing symptom” (23.3%), characterized by gradually worsening moderate depressive symptoms. These trajectory patterns were in line with previous studies of NKDs. Studies consistently reported that the severity of NKDs' depressive symptoms increased significantly according to the period of settlement in South Korea (20, 31), but most of the participants did not report clinically significant depressive symptoms. Consistent with the findings that most NKDs exhibit mild depressive symptoms, several refugee studies also showed that most people do not report pathological symptoms after resettlement (53). Similarly, a recent refugee study, using latent transitional analysis, found that 61–68% of the participants did not report PTSD or depression (32). Like previous studies, in this study, more than half of the NKDs did not show serious pathology, and only some showed patterns indicating moderate depressive symptoms (13).

According to the results derived from the comparison between characteristics of trajectory groups at baseline, the second main finding was that the proportion of women was higher in the high/increasing symptom group than in the low/stable symptom group. Specifically, approximately 94% individuals in the high/increasing symptom group were women, confirming previous findings. For instance, according to a meta-analysis on NKDs, many scholars found gender differences in depressive symptoms (3). Women are known to be more vulnerable to emotional problems and the prevalence of major depressive disorder is also known to be higher in women (14). Therefore, because most of this study's NKDs were women and exhibited the risk of worsening depressive symptoms while adapting to South Korean society, there is a need to pay particular attention to women's mental health. In the present study, members of the high/increasing symptom group did not belong to a significantly high age or low educational background. Accordingly, previous research reported that the relationship between participants' age or educational years and mental health problems was inconsistent (3). However, it is possible that the effect of age was not significant because this study's age range was too wide, ranging from 18 to 73 years. Additionally, in a previous follow-up study, educational background acted as a protective factor in the initial settlement process, but the relationship between depression and academic background was not significant after 3 years (20). The long-term effect of the educational year was considered unclear.

The third main finding was that the high/increasing symptom group had a significantly higher proportion of unemployed individuals, but there was no significant difference in the proportion of low-income families. According to a survey conducted by the North Korean Refugee Support Foundation, the shorter the period of residence in South Korea, the higher the proportion of low-income families; the longer the period of residence, the higher the average annual income (13). Therefore, because this study targeted NKDs who entered the country within the first 3 years of the first wave, it is possible that most individuals were in the low-income class, regardless of the degree of depression. As one of the main reported reasons for dissatisfaction with the South Korean society was high competitiveness (13), many individuals have difficulty adapting to South Korean competition-based systems in the employment process. Thus, to successfully find a job in the initial resettlement period, NKDs may require some psychological and social resources to quickly adapt in South Korea. A stable professional life is known to contribute significantly to the improvement of preexisting mental health problems and life satisfaction (54). Therefore, rather than only providing subsidies, offering steady vocational education and various job opportunities can help NKDs appropriately adapt to a competitive society.

The fourth main finding was that the proportion of children in the family was higher in the high/increasing symptom group than in the low/stable symptom group. Among the indicators related to the social environment, this group had a lower proportion of mental health service utilization, but there was no significant difference in the degree of perceived social support and proportion of religious life. Although research has reported that living in South Korea with family members causes less psychiatric problems (21), according to Kim (55), NKDs who have children experience more depression or somatic symptoms. Based on the Hana Center's published statistics, more than half of the respondents found child education costs to be a burden on households. Thus, one can infer that economic pressure may have an effect on increasing depression. In addition, it was found that due to environmental changes, various difficulties in raising children were observed (56). The child-centered culture of South Korean society can cause stress and confusion in role performance for parents. In the process of settlement, parents face secondary difficulties such as conflicts with their children and their adaptation to school. Research has also indicated that religious tendencies or activities did not have a significant correlation with depressive symptoms (57). Social supports may be a protective factor for depression in adolescents, when connectivity with peers is considered the most important (8, 58); however, in adulthood, practical help from mental health experts is more important than any other factor.

The fifth main finding was that the high/increasing symptom group had higher levels of clinically related psychological indicators and lower levels of adaptive psychological indicators. Specifically, there were significant differences in lifetime suicidal ideation, traumatic experience and loneliness, and resilience. Moreover, the high/increasing symptom group was characterized by a significantly higher proportion of all mental disorders, except for alcohol-related disorders. In this regard, previous research showed that maladaptive characteristics, such as low resilience, can predict psychological symptoms (59), consistent with this study's findings. Additionally, most clinical indicators increased significantly, indicating that psychiatric problems and worsening depressive symptoms were correlated. This suggests that depressive symptoms can be a major indicator of NKDs' mental health. According to studies conducted with the same cohort, this group is known to be psychologically vulnerable, with higher major psychiatric diagnoses and suicidal ideation compared to the general population (5). Therefore, it is important to select high-risk mental health groups and conduct priority interventions.

Logistic analysis was performed to derive variables that could predict the high/increasing symptom trajectory. Among sociodemographic and environmental variables, being a woman and having less experience with receiving mental health services resulted in a higher likelihood of worsening depressive symptoms. In the defection process, women often become victims of psychological trauma and sexual assaults compared to men (60). As they have lived in a society with enforced gender role stereotypes, their social resources, such as basic skills and job search experience, are often relatively insufficient. In other words, more efforts are needed for adaptation among women than among men. Despite many vulnerabilities, women are more receptive to new sociocultural values than men (61). Therefore, early interventions are necessary and should focus on women's depression at the moment of entry. According to previous studies on using mental health services, more NKDs responded with “do not know” when asked about psychological disorders compared to South Koreans, and negative perceptions of mental disorders were widespread (49). In addition, the lower the health literacy, which implies one's ability to accurately understand the information necessary to make appropriate health-related decisions, the lower one's experience with preventive health care systems (62). Therefore, basic mental health education and interventions that change one's perception of mental health should be improved and continued. Nevertheless, according to a number of refugee studies, attention is being paid to the positive resources of refugees (63). As a result of conducting value-based counseling on refugees, enhancing the client's self-efficacy and ultimately demonstrating their inherent resilience, it was found that most psychological indicators, such as depression and anxiety, improved in comparison to the control group (64). Therefore, it is crucial to create a strength-based therapeutic environment that helps NKDs receive direct support when necessary.

Furthermore, mental disorders, such as generalized anxiety disorder, social phobia, and alcohol dependence, predicted worsening depressive symptoms. Thus, anxiety levels precede the worsening of depressive symptoms. According to previous refugee-related studies (65), trauma survivor groups exhibit high proportions of major depressive and social anxiety disorders. Studies on war survivors showed that social anxiety hinders one's ability to cultivate satisfying social relationships and prevents exposure to situations that cause positive emotional experiences, slowing recovery as a result (66). In addition, the biggest difficulty in supporting NKDs is their “anxiety and distrust” (67). Specifically, it was difficult for the NKDs to honestly reveal themselves and form relationships, due to their negative cognitions related to trust. However, even if interpersonal relationships are established, they seem to be vulnerable to negative feedback. For this reason, it is crucial to consider NKDs' anxiety levels, as one's psychological adaptation to South Korean society can be defined as a mental state free of anxiety when conducting daily activities. In addition, in the case of alcohol dependence, the difference between groups was not significant in the *t*-test, but it was found to be a significant predictor in the logistic regression. Therefore, even if the influence of other mixed variables, such as comorbid diseases, is excluded, a history of alcohol dependence can affect the deterioration of depressive symptoms. Previously conducted in-depth interviews with NKDs showed that quitting alcohol consumption was an important factor for improving depression (23). Evidently, maladaptive coping methods, such as alcohol consumption, can have adverse effects on one's physical health and psychosocial environment.

Contrary to expectations, in this study, PTSD did not significantly predict worsening depressive symptoms. However, although the diagnosis of PTSD was not significant, the prevalence (12.3% vs. 26.6%) and severity of PTSD symptoms (IES-R-NK: 25.54 vs. 40.28) were reported to be higher in the high/increasing symptom group than in the low/stable symptom group. As shown in previous studies, the prevalence of PTSD among NKDs is characterized by a higher rate than that of other disorders (5). In 2007, the prevalence of full PTSD was reported as 26.15% and that of partial PTSD was reported as 50.66% (68). In this study, it was also found that the lifetime prevalence rate of PTSD was 15.3%, higher than that for South Koreans at 1.9%. Therefore, it is thought that NKDs' PTSD is more prevalent in comparison to general groups. Furthermore, it is known that actual PTSD diagnosis tends to differ according to the degree of exposure to traumatic events (5, 69). In PTSD, the prevalence rate is reported differently depending on the characteristics of the target, measurement tools, and evaluation methods, so symptoms should be judged from various angles (68). For example, in the self-report test, the IES-NK score of the high/increasing symptom group was 40.28 and that of the stable/low symptom group was 25.54. Considering that the cut-off is suggested to be about 24/25 points (42), each group may be considered to be accompanied by PTSD and major depressive disorder based on the self-report score. Therefore, in this study, although PTSD diagnosis was not a significant factor predicting the longitudinal progress of NKDs' depressive symptoms, it should be considered important when performing diagnostic evaluation (29, 69).

Limitations of this study must be addressed. First, the CES-D-NK tool was not used in the T2 and T4 waves. Since the degree of depression could not be determined at both points, there is a possibility of some limitations in constructing a sophisticated trajectory. Second, most of the scales were based on self-reporting, and some were not translated into North Korean. Thus, symptom reporting may be limited, and due to the characteristics of NKDs, sensitive information was not actively provided, which could reduce reliability. Third, as certain questions encompassed a variety of factors, such as those related to using health services or religious experience, they may not accurately reflect the individuals' characteristics. Fourth, the participants were heterogeneous. While the participants were registered at the Hana Center after entering South Korea within the last 3 years, they may have less similarities in terms of their age or baseline depression levels. In other words, there were limitations to interpreting the findings by applying latent mixed modeling. Fifth, it is necessary to consider the specificity of NKDs belonging to the Hana Center. A group with a higher socioeconomic status than the general North Korean population may be included, as a large amount of money is required for migrating to South Korea. In addition, generalizability limitations are possible, considering that an emotionally stable group may have been included (e.g., individuals with relatively little exposure to traumatic experiences in the repatriation process). Moreover, the fact that most of the participants were women acts as a limitation in generalizing the results. Sixth, as indicators related to direct social adaptation levels, such as work or spare time and coping abilities, were not included, it was hard to clearly describe the relationship between the severity of mood symptoms and actual adaptation. Furthermore, questions measuring pre-migration factors were not included in the data collection process, so there was a limitation in exploring PTSD symptoms comprehensively. Finally, since latent class mixed analysis is a method of dividing groups based on probability, the properties of each group may not be sufficiently reflected. In future studies, it may be helpful to diversify materials to increase the accuracy of symptom reporting. In addition, it is possible to search for trajectories related to the anxiety symptom for each wave. Furthermore, by examining the difference in the depressive trajectories between the general population and NKDs, it is possible to identify specificity of depression within a minority group and propose customized policies.

Overall, this study explored the trajectory of depression, measured by CES-D-NK, in 300 NKDs, with 76.7% being classified as low/stable and 23.2% as high/increasing. Predictors of the high/increasing symptom group were being female and having a low use of mental health services, alongside a history of generalized anxiety disorder, social phobia, and alcohol dependence. Accordingly, attention should be paid to NKDs' anxiety levels and maladaptive drinking habits, but the top priority should be creating an environment in which mental health services are conveniently available. Although PTSD was not a significant predictor, there should be an appropriate consideration of PTSD symptoms. Providing relevant basic information to devise government-level policies, by selecting high-risk groups that require clinical treatment, and increasing NKDs' understanding of mental health can also be a meaningful step.

## Data availability statement

The raw data supporting the conclusions of this article will be made available by the authors, without undue reservation.

## Ethics statement

The studies involving human participants were reviewed and approved by the Institutional Review Board of Samsung Medical Center (SMC 2015-05-042-002). The patients/participants provided their written informed consent to participate in this study.

## Author contributions

DK participated in the study design, data analysis, wrote the first manuscript draft including the Introduction, Methods, Results, and Discussion. SP participated in data curation. JA, MK, and HC participated in the study design. JH participated in whole study design and supervised the data analyses and interpretations. All authors read and approved the final manuscript.

## Funding

This work was supported by the Korea Healthcare Technology R&D project, Ministry of Health and Welfare, Republic of Korea (HL19C0018).

## Conflict of interest

The authors declare that the research was conducted in the absence of any commercial or financial relationships that could be construed as a potential conflict of interest.

## Publisher's note

All claims expressed in this article are solely those of the authors and do not necessarily represent those of their affiliated organizations, or those of the publisher, the editors and the reviewers. Any product that may be evaluated in this article, or claim that may be made by its manufacturer, is not guaranteed or endorsed by the publisher.
